# Effect of interval between oocyte retrieval and resuscitation embryo transfer on pregnancy outcomes

**DOI:** 10.3389/fmed.2022.1081782

**Published:** 2023-01-04

**Authors:** Qi Wan, Ming-Xing Chen, Xuejiao Wang, Li Tan, Hui-Jun Yu, Xing-Yu Lv, Zhao-Hui Zhong, Xiao-Jun Tang, Yu-Bin Ding, Min Xia, Yuan Li

**Affiliations:** ^1^The Reproductive Center, Chengdu Jinjiang Hospital for Women’s and Children’s Health, Chengdu, Sichuan, China; ^2^Department of Obstetrics and Gynecology, West China Second Hospital, Sichuan University, Chengdu, China; ^3^Key Laboratory of Birth Defects and Related Diseases of Women and Children, Ministry of Education, Sichuan University, Chengdu, China; ^4^Joint International Research Laboratory of Reproduction and Development of the Ministry of Education of China, School of Public Health, Chongqing Medical University, Chongqing, China; ^5^Department of Epidemiology, School of Public Health, Research Center for Medicine and Social Development, Innovation Center for Social Risk Governance in Health, Chongqing Medical University, Chongqing, China; ^6^Department of Gynecology, Chongqing Hospital of Traditional Chinese Medicine, Chongqing, China

**Keywords:** immediate frozen embryo transfer, delayed frozen embryo transfer, live birth rate, clinical pregnancy, pregnancy outcome

## Abstract

**Objectives:**

Resuscitation transfer of embryos after elective cryopreservation has been widely applied in *in vitro* fertilization-embryo transfer (IVF-ET) therapy for human infertility or sterility owing to higher embryo implantation rates. This method separates oocyte retrieval from embryo transfer. The optimal time for frozen embryo transfer (FET) remains unknown. Therefore, this study mainly compares the advantages and disadvantages of delayed FET and immediate FET through retrospective analysis.

**Methods:**

We analyzed real world data of patients who underwent resuscitation transplantation between October 2019 and July 2021 at the Reproductive Center of Chengdu Jinjiang Hospital for Women’s and Children’s Health. Propensity score matching was applied to control potential confounding factors. A total of 5,549 patients who received at least one FET were analyzed. Patients undergoing transplantation within 60 days of oocyte retrieval were included in the immediate FET group (*n* = 1,265) and those undergoing transplantation > 60 days after retrieval were included in the delayed FET group (*n* = 4,284).

**Results:**

Live birth rates between the two groups were comparable (45.25% vs. 45.76%, *p* = 0.757). Moreover, no difference was observed in the rates of biochemical pregnancy (64.50% vs. 66.80%), clinical pregnancy (55.24% vs. 56.83%), ectopic pregnancy (1.47% vs. 1.39%), early miscarriage (14.41% vs. 16.20%), late miscarriage (2.21% vs. 2.09%), singleton premature delivery (16.67% vs. 18.29%), and neonatal deformity (1.97% vs. 1.80%). After stratifying the patients based on the type of embryo transferred, number of embryos transferred, FET protocol, and good prognosis criteria, live birth rates remained comparable between the two groups (*p* > 0.05).

**Conclusion:**

Pregnancy outcomes were comparable between the immediate and delayed FET groups.

## 1. Introduction

Controlled ovarian hyperstimulation (COH) is one of key processes in assisted reproductive technology (ART) therapy. COH may lead to ovarian hyperstimulation syndrome (OHSS), cause endometrial synchronicity and other adverse factors that could reduce pregnancy rates, such as elevated progesterone and tubal effusion. Nearly half of the patients receiving COH undergo resuscitation transplantation after embryo freezing (1–4). Patients undergoing *in vitro* fertilization (IVF) are eager to get pregnant with shortened interval between egg retrieval and transplantation. However, there is no uniform regulation regarding the timing of post-COH resuscitation transplantation (5, 6). However, since the ovaries are enlarged during COH and the risk of ovarian torsion are increased, physicians of reproductive medicine tend to start embryo resuscitation and transplantation preparations until at least the second menstrual cycle.

In addition, more and more young cancer patients accept fertility preservation services at present since they receive certain types of cancer surgery which lead to removal of organs needed for a pregnancy, and certain therapy might increase hormone levels or cause damage to a female’s eggs (7). Previous studies have not come to a definitive conclusion on how immediate or delayed resuscitation will benefit pregnancy rates (8, 9). Additionally, many of the retrospective studies on this issue were conducted in years ago, and the results of these studies may be biased against changes in the existing COH protocols (1, 10, 11). Therefore, owing to the advantage of large data volumes at our hospital, we retrospectively analyzed the clinical data of patients who underwent COH and resuscitation transplantation from October 2019 to August 2021. We compared clinical data from first, second, and successive menstrual cycle resuscitations thereafter involving full embryo freezing.

## 2. Materials and methods

### 2.1. Patients

This retrospective cohort study reviewed the data of patients who underwent IVF/intracytoplasmic sperm injection (ICSI) between October 2019 and July 2021 at the Reproductive Center of Chengdu Jinjiang Hospital for Women’s and Children’s Health, Sichuan, China. This study was approved by the Ethics Committee of the Chengdu Jinjiang Hospital for Women’s and Children’s Health. And all methods were performed in accordance with the relevant guidelines and regulations. The informed consent was obtained from all participants of the study.

Patients aged 20–38 years who underwent IVF/ICSI cycles, patients who froze all embryos, and patients who subsequently received FET after failure of fresh embryo transplantation were included in the study. Further, patients who underwent rescue ICSI cycles, with an endometrial thickness of <8 mm before embryo transfer, and patients with endometriosis, genital malformation, or uterine abnormality were excluded.

### 2.2. COH protocols

Four ovulation stimulation protocols were used. (I) The antagonist protocol, involving ovulation induction with follicle stimulating hormone (FSH) (Gonal-F, Merck Serono, Puregon, Organon) from days 2–5 of menstruation, followed by gonadotropin releasing hormone antagonist (GnRH-a) (Cetrorelix, Merck Serono, or Orgalutran Organon) at a daily dose of 0.25 mg, which was commenced when the largest follicle exceeded 12–14 mm. (II) The long ovulation stimulation protocol, involving 3.75 mg GnRH-a (Triptorelin, Ferring) injections on the 2nd–5th day of menstruation and FSH ovulation induction after 28–30 days. (III) The luteal phase improvement long protocol, involving 0.1 mg GnRH-a (Triptorelin, Ferring) injection during the luteal phase followed by FSH administration starting on the 3rd day of menstruation to initiate ovulation. (IV) The micro stimulation protocol, involving administration of FSH or Menotrophins (Lebaode, LiZhu) on the 2nd–3rd day of menstruation was used to promote egg excretion. Urinary human chorionic gonadotropin 2000–6000 and GnRH-a 0.2 mg or recombinant human choriogonadotropin alfa (AiZe, Merck Serono, Darmstadt, Germany) were then injected when the target follicle was 18–20 mm. Egg retrieval was carried out 36–38 h after triggering, and 90 mg/d of crinone (Merck Serono, Darmstadt, Germany) and 20 mg/d of dydrogesterone (Abbott Biologicals, Beijing, China) were administered as luteal support after egg retrieval. All frozen embryos were vitrified with open system.

### 2.3. Endometrial preparation

Patients who underwent transplantation within 60 days of oocyte retrieval were included in the immediate FET group and those who underwent transplantation > 60 days after retrieval were included in the delayed FET group. The endometrial preparation program included natural cycle, hormone replacement cycle, ovulation-promoting cycle, and down-regulation cycle. The natural cycle was determined by monitoring follicular development using transvaginal ultrasonography and hormone levels. For the hormone replacement cycle, on days 2–4 of the menstrual cycle, 4 mg estradiol valerate tablets (Progynova, Berlin, Germany) were administered daily for 10 days. For the ovulation-promoting cycle, on days 2–4 of menstruation, 2.5–5 mg of letrozole, 20–40 mg of tamoxifen, or 50 mg of clomiphene was administered for 5 days. The down-regulation cycle involved injection of 3.75 mg GnRH-a (Triptorelin, Ferring) on days 2–3 of menstruation.

Luteal transformation was achieved with 90 mg/d of crinone or 600 mg/d of soft capsule progesterone (Cyndea Pharma, S.L.) and 40 mg/d of dydrogesterone, after the endometrium reached 8 mm thickness. Cleavage-stage embryos or blastocysts were transferred 3–5 days after transformation. Luteal support was provided after embryo transfer.

### 2.4. Outcomes

The primary outcome was the live birth rate, defined as the delivery of a living baby at ≥28 weeks of pregnancy after the first embryo transfer. The secondary outcomes were the rates of biochemical pregnancy, clinical pregnancy, ectopic pregnancy, early miscarriage, late miscarriage, singleton premature delivery, and neonatal deformity.

### 2.5. Statistical analysis

Propensity score matching (PSM) was used to make the baseline characteristics between the immediate and delayed FET groups balanced and comparable. The variables for PSM included female age, FSH, progesterone, fertilization method, COS protocol, FET protocol, the number of top-quality embryos transferred, and the type of embryo transferred. The (1:3) nearest neighbor caliper matching method without replacement was used to match data between the two groups, and the caliper was 0.05. The Kolmogorov–Smirnov test and the Shapiro–Wilk test were used to test the normality of the variables. Continuous variables are presented as mean ± SD or median (IQR). Categorical variables are presented as numbers and percentages. Continuous variables were compared between the groups using the *t*-test or Mann–Whitney *U* test. Categorical variables were compared using the Chi-square test. Multiple logistic regression models, odds ratio (OR), and 95% confidence intervals (CI) were calculated after adjusting for confounders. A *P*-value < 0.05 was considered statistically significant. All analyses were performed using SPSS software, version 25.0 and R software version 3.3.0.

## 3. Results

### 3.1. Demographics

All demographic data are shown in Table 1. A total of 5,549 patients who received at least one FET were analyzed in the study. The immediate FET group consisted of 1,265 patients, and the delayed FET group consisted of 4,284 patients. The interval between oocyte retrieval and embryo recovery was significantly shorter in the immediate FET group than in the delayed FET group [days: 34 (30–56) vs. 83 (67–111)]. Additionally, there were significant differences in maternal age, basal FSH, basal P, fertilization method, COS protocol, FET protocol, the number of top-quality embryos transferred, and the type of embryo transferred between the two groups (*p* < 0.05). After PSM, a total of 1,231 immediate FET patients were successfully matched to 3,280 delayed FET patients. The interval between oocyte retrieval and embryo recovery was still significantly shorter in the immediate FET group than in the delayed FET group [days: 30 (27, 33) vs. 69 (59, 94)]. After PSM, there were no significant differences in maternal age, basal FSH, basal progesterone, fertilization method, FET protocol, and number of top-quality embryos transferred between the two groups (*p* > 0.05). However, the COS protocol and type of embryo transferred were significantly different between the two groups (*p* < 0.05).

**TABLE 1:** Comparison of baseline data between the groups before and after propensity matching.

	Before PSM			After PSM		
	Immediate FET group	Delayed FET group	*P*-value	Immediate FET group	Delayed FET group	*P*-value
No.	1,265	4,284		1,231	3,280	
Interval days	34 (30, 56)	83 (67, 111)	<0.001	30 (27, 33)	69 (59, 94)	<0.001
Age	30 (27, 33)	31 (28, 33)	<0.001	30 (27, 33)	30 (28, 33)	0.846
BMI	21.67 (19.73, 24.00)	21.48 (19.82, 23.81)	0.469	21.52 (19.83, 23.88)	21.60 (19.71, 23.81)	0.384
Infertility years	3 (1, 4)	3 (2, 5)	0.055	3 (2, 5)	3 (2, 5)	0.152
FSH	7.42 (6.28, 8.75)	7.20 (6.21, 8.40)	<0.001	7.31 (6.26, 8.65)	7.25 (6.26, 8.45)	0.061
LH	4.52 (3.29, 6.17)	4.43 (3.23, 6.14)	0.294	4.52 (3.31, 6.34)	4.54 (3.34, 6.35)	0.484
E2	45.00 (34.00, 58.00)	44.0 (34.00, 57.00)	0.204	44 (33, 57)	43 (33, 56)	0.259
P	0.59 (0.40, 0.89)	0.57 (0.38, 0.83)	0.008	0.60 (0.40, 0.88)	0.58 (0.39, 0.85)	0.210
AFC	18 (11, 26)	89 (12, 26)	0.136	19 (12, 27)	19 (13, 27)	0.075
AMH	4.29 (2.32, 6.76)	4.37 (2.59, 6.63)	0.132	4.49 (2.61, 7.03)	4.61 (2.74, 6.94)	0.095
FET day endometrial thickness	9.00 (8.50, 10.50)	9.50 (8.50, 10.50)	0.464	9.5 (8.5, 10.5)	9.5 (8.5, 10.5)	0.949
Types of infertility			0.246			0.748
Primary	666 (52.65%)	2176 (50.79%)		645 (52.40%)	1701 (51.86%)	
Secondary	599 (47.35%)	2108 (49.21%)		586 (47.60%)	1579 (48.14%)	
Fertilization method			0.049			0.733
IVF	1051 (83.08%)	3454(80.63%)		1022 (83.02%)	2709 (82.59%)	
ICSI	214(16.92%)	830 (19.37%)		209 (16.98%)	571 (17.41%)	
COS protocol			<0.001			0.001
Antagonist protocol	775 (59.17%)	2535 (61.26%)		774 (62.88%)	2093 (62.88%)	
Long protocol	262 (20.71%)	1063 (24.81%)		262 (21.28%)	753 (22.96%)	
Luteal phase improvement long protocol	55 (4.35%)	256 (5.98%)		55 (4.47%)	158 (4.82%)	
PPOS	131 (10.36%)	169 (3.94%)		98(7.96%)	154 (4.70%)	
Others	42 (3.32%)	261 (6.09%)		42(3.41%)	122 (3.72%)	
Endometrial preparation program			<0.001			0.863
HRT	1141 (90.20%)	3418 (79.79%)		1109 (90.09%)	2935 (89.48%)	
DRC	16 (1.26%)	324 (7.56%)		16 (1.30%)	53 (1.62%)	
OPC	37 (2.92%)	251 (5.86%)		37 (3.01%)	105 (3.20%)	
NC	71 (5.61%)	291 (6.79%)		69 (5.61%)	187 (5.70%)	
NET			0.091			0.496
1	436 (34.47%)	1588 (37.07%)		422 (34.28%)	1160 (35.37%)	
2	829 (65.53%)	2696 (65.53%)		809 (65.72%)	2120 (64.63%)	
HQEN			<0.001			0.449
0	363 (28.70%)	1521 (35.50%)		360 (29.24%)	1023 (31.19%)	
1	529 (41.82%)	1622 (37.80%)		515 (41.84%)	1330 (40.55%)	
2	373 (29.49%)	1141 (26.63%)		356 (28.92%)	927 (28.26%)	
ET			<0.001			<0.001
Cleaved-embryo	240 (18.97%)	458 (10.69)		212 (17.22%)	410 (12.50%)	
Blastocyst	1025 (81.03%)	3826 (89.31%)		1019 (82.78%)	2870 (87.50%)	

BMI, body mass index; HRT, hormone replacement cycle; DRC, down-regulation cycle; OPC, ovulation-promoting cycle; NC, natural cycle; NET, number of embryos transferred; HQEN, high-quality embryos number; ET, embryo transfer type.

### 3.2. Pregnancy outcomes

Before PSM, the live birth rate was not significantly different between the immediate FET and the delayed FET groups (45.22 vs. 45.38%, *p* = 0.920) (Table 2). Additionally, after PSM the live birth rates between the groups remained comparable (45.25 vs. 45.76%, *p* = 0.757) (Table 2). Moreover, no significant differences were observed in the rates of biochemical pregnancy (64.50 vs. 66.80%), clinical pregnancy (55.24 vs. 56.83%), ectopic pregnancy (1.47 vs. 1.39%), early miscarriage (14.41 vs. 16.20%), late miscarriage (2.21 vs. 2.09%), singleton premature delivery (16.67 vs. 18.29%), and neonatal deformity (1.97 vs. 1.80%) between the immediate and delayed FET groups, respectively.

**TABLE 2:** Comparison of pregnancy outcomes between the immediate and delayed groups before and after PSM.

	Before PSM			After PSM		
	Immediate FET group	Delayed FET group	*P*-value	Immediate FET group	Delayed FET group	*P*-value
LBR	45.22% (572/1,265)	45.38% (1,944/4,284)	0.920	45.25% (557/1,231)	45.76% (1,501/3,280)	0.757
BPR	64.51% (816/1,265)	66.59% (2,849/4,284)	0.187	64.50% (794/1,231)	66.80% (2,191/3,280)	0.146
LPR	55.26% (699/1,265)	56.47% (2,419/4,284)	0.446	55.24% (680/1,231)	56.83% (1,864/3,280)	0.338
EPR	1.43% (10/699)	1.28% (31/2,419)	0.761	1.47% (10/680)	1.39% (26/1,864)	0.886
EMR	14.59% (102/699)	16.62% (402/2,419)	0.200	14.41% (98/680)	16.20% (302/1,864)	0.272
LMR	2.15% (15/699)	1.90% (46/2,419)	0.681	2.21% (15/680)	2.09% (39/1,864)	0.860
SPDR	16.27% (69/424)	17.01% (234/1,376)	0.725	16.67% (69/414)	18.29% (193/1,055)	0.464
NDR	1.92% (11/572)	1.49% (29/1,944)	0.469	1.97% (11/557)	1.80% (27/1,501)	0.792

LBR, live birth rate; BPR, biochemical pregnancy rate; PR, clinical pregnancy rate; EPR, ectopic pregnancy rate; EMR, early miscarriage rate; LMR, late miscarriage rate; SPDR, singleton premature delivery rate; NDR, neonatal deformity rate; PSM, propensity score matching.

The COS protocol and type of embryo transferred are displayed in Table 3. The interval between oocyte retrieval and embryo recovery had no significant effect on pregnancy outcomes. The findings in Table 3 after PSM are consistent with the results from multivariate regression analysis adjusted for potential confounding factors, including maternal age, basal FSH, basal P, fertilization method, COS protocol, FET protocol, number of top-quality embryos transferred, and type of embryo transferred.

**TABLE 3:** Multivariate logistic regression analysis of pregnancy outcomes for immediate FET and delayed FET.

	Before PSM		After PSM	
	Adjusted OR (95% CI)	*P*-value	Adjusted OR (95% CI)	*P*-value
LB	1.033 (0.907, 1.178)	0.622	1.016 (0.889, 1.160)	0.819
BP	0.972 (0.848, 1.115)	0.687	0.940 (0.818, 1.080)	0.383
CP	0.994 (0.872, 1.133)	0.926	0.971 (0.850, 1.110)	0.668
EP	0.930 (0.443, 1.955)	0.849	0.958 (0.456, 2.011)	0.910
EM	0.873 (0.684, 1.114)	0.274	0.854 (0.666, 1.095)	0.212
LM	1.037 (0.565, 1.901)	0.907	1.043 (0.569, 1.909)	0.892
SPD	0.993 (0.734, 1.343)	0.962	0.911 (0.673, 1.234)	0.547
NDR	1.187 (0.577, 2.442)	0.641	1.093 (0.536, 2.228)	0.806

LR, live birth; BP, biochemical pregnancy; CP, clinical pregnancy; EP, ectopic pregnancy; EM, early miscarriage; LM, late miscarriage; SPD; singleton premature delivery; NDR, neonatal deformity rate; PSM, propensity score matching.

### 3.3. Live birth outcomes by stratification analysis

To compare the live birth rates between the immediate and delayed FET groups in patients with different characteristics, we carried out further analysis by stratifying the patients according to the type of embryo transferred, number of embryos transferred, FET protocol, and criteria for good prognosis (AMH > 1.1 ng/ml and AFC > 8) (Tables 4, 5). After PSM, live birth rates were comparable among the groups for each stratification (*p* > 0.05). Additionally, after adjusting for the COS protocol and type of embryo transferred, multivariate logistic analysis on the four stratified groups revealed no significant correlation between oocyte retrieval and embryo recovery with live birth rates (Tables 4, 5).

**TABLE 4:** Stratified analysis comparing the live birth rate between the immediate FET and delayed FET groups.

	Before PSM			After PSM		
	Immediate FET group	Delayed FET group	*P*-value	Immediate FET group	Delayed FET group	*P*-value
**ETT**						
Cleaved	37.50% (90/240)	32.75% (150/458)	0.210	36.79% (78/212)	30.98% (127/410)	0.144
Blastocyst	47.02% (482/1,025)	46.89% (1,794/3,826)	0.939	47.01% (479/1,019)	47.87% (1,374/2,870)	0.634
**NT**						
1	34.86% (152/436)	34.13% (542/1,588)	0.776	34.60% (146/422)	33.71% (391/1,160)	0.741
2	50.66% (420/829)	52.00% (1,402/2,696)	0.500	50.80% (411/809)	52.36% (1,110/2,120)	0.451
**EPP**						
HRT	45.49% (519/1,141)	45.49% (1,555/3,418)	0.996	45.63% (506/1,109)	45.79% (1,344/2,935)	0.925
DRC	56.25% (9/16)	43.52% (141/324)	0.317	56.25% (9/16)	32.08% (17/53)	0.080
OC	45.95% (17/37)	45.82% (115/251)	0.988	45.95% (17/37)	48.57% (51/105)	0.783
NC	38.03% (27/71)	45.70% (133/291)	0.243	36.23% (25/69)	47.59% (89/187)	0.105
AMH > 1.1 and AFC > 8	47.30% (491/1,038)	46.59% (1,755/3,767)	0.683	47.29% (489/1,034)	47.36% (1,375/2,903)	0.968

ETT, embryo transfer type; NT, number of embryos transferred; EPP, endometrial preparation program; HRT, hormone replacement cycle; DRC, down-regulation cycle; OC, ovulation-promoting cycle; NC, natural cycle.

**TABLE 5:** Multivariate logistic regression analysis of live birth rates for immediate FET and delayed FET.

	Before PSM		After PSM	
	Adjusted OR (95% CI)	*P*-value	Adjusted OR (95% CI)	*P*-value
**ETT**				
Cleaved	1.321 (0.932, 1.873)	0.118	1.339 (0.941, 1.905)	0.105
Blastocyst	0.991 (0.861, 1.142)	0.905	0.969 (0.840, 1.119)	0.671
**NT**				
1	1.088 (0.863, 1.373)	0.476	1.078 (0.850, 1.366)	0.537
2	1.003 (0.854, 1.179)	0.968	0.981 (0.832, 1.156)	0.821
**EPP**				
HRT	1.179 (1.036, 1.342)	0.577	1.027 (0.893, 1.181)	0.712
DRC	2.091 (0.710, 6.155)	0.181	2.934 (0.817, 10.533)	0.099
OC	1.141 (0.543, 2.398)	0.727	0.956 (0.424, 2.157)	0.913
NC	0.645 (0.362, 1.147)	0.135	0.607 (0.335, 1.101)	0.101
AMH > 1.1 and AFC > 8	1.041 (0.904, 1.200)	0.575	1.014 (0.879, 1.170)	0.852

ETT, embryo transfer type; NT, number of embryos transferred; EPP, endometrial preparation program; HRT, hormone replacement cycle; DRC, down-regulation cycle; OC, ovulation-promoting cycle; NC, natural cycle.

## 4. Discussion

Patients undergoing embryo transplantation during fresh cycles have elevated estrogen and progesterone levels and unpredictable OHSS may occur. Although, there are some studies suggest that the administration of a rescue double GnRH antagonist dose at 1 day before hCG trigger may represent a safe alternative preventive strategy for preventing early OHSS without affecting the reproductive outcomes (12). However, more studies are pointing to embryo transfer in resuscitation cycles to improve clinical pregnancy rates and live birth rates and to reduce the occurrence of OHSS (13, 14). There are many randomized controlled trials comparing the advantages and disadvantages of conventional whole embryo freezing and fresh cycle transfer strategies (15). Although there is no favorable evidence to prove that whole embryo freezing has better advantages, clinicians are increasingly using this method (16). A recent review mentioned that in the long-term follow-up of newborns, it was found that the perinatal incidence rate and neonatal congenital malformation rate of patients undergoing FET were similar to those of patients undergoing fresh embryo transfer, and in some specific nervous systems, newborns who had frozen embryo pregnancy even had better cognitive function. Therefore, it is suggested that frozen embryo resuscitation transfer is a safe and reliable choice in clinical practice (17).

If whole embryo freezing is selected despite the risks of OHSS and increase of progesterone during COH, it is suggested that the preparation for resuscitation and transplantation can start immediately. However, patients may need to prolong the time before FET, owing to several reasons, such as endometrial abnormalities and hydrosalpinx. Of note, the optimal time to perform resuscitation and transfer after embryo freezing is yet unclear. Existing studies are primarily based on retrospective analysis, and high quality prospective randomized controlled studies are needed.

In previous retrospective studies, they found that immediate FET had higher live birth rates than delayed FET (18, 19). However, other studies concluded that immediate and delayed FET did not differ in clinical pregnancy and live birth rates (20–22). In those studies, the use of natural cycles in endometrial preparation protocols was excluded from the cycles due to ovulatory disturbances because of the impact of COH. Our study included natural cycle endometrial preparation, which means data is more comprehensive; however, the immediate FET group had fewer natural cycles than the delayed FET group, that might due to the patients in the immediate FET group have ovulation disorders and were not suitable for the natural cycle scheme. In the immediate FET group, the hormone replacement cycle was not affected by ovarian cysts. Additionally, hormone replacement can inhibit the growth of follicles, thereby reducing physiological cysts after egg retrieval. It is also the preferred option. We did not record the cycles that were canceled due to ovulation disorders. Our study concluded that immediate FET and delayed FET showed comparable live birth rates for different embryo types, embryo numbers, and different endometrial preparation protocols, which is consistent with the findings of other studies (1, 6). Therefore, there is no evidence to support the necessity for routine delay of at least one menstrual cycle after IVF/ICSI before FET.

However, patients with breast cancer or leukemia need fertility preservation before tumor therapy, embryo or egg freezing is their main choice for fertility preservation and they have to delayed FET. A retrospective study showed that 43% of breast cancer patients decided to preserve their fertility (7), and another study shows that whether performing COH before, or ART following anticancer treatment in young women with breast cancer does not seem to be associated with detrimental prognostic effect in terms of breast cancer recurrence, mortality or event-free survival (23). Therefore, the delayed resuscitation is also a good choice for tumor patients. Egg freezing is similar to embryo freezing, since 2013, egg cryopreservation has no longer been considered experimental by the American, clinical application faces some ethical challenges (24, 25), such as the effect on tumor recurrence of patients. For some patients, such as patients with endometrial cancer, there have been some studies on molecular level to find out whether the patients are suitable for fertility preservation and whether these treatments have an impact on the prognosis of patients (26). These studies are very meaningful for guiding our treatment.

Now the embryo freezing has been well development and utilized in IVF therapy or fertility preservation, and the frozen embryo is mainly carried out through open and closed carriers. Some scholars believe that the closed carrier for embryo freezing can reduce the risk of sample pollution, and the closed vitrification system may be the future development trend (27). However, although it is theoretically believed that there is a risk of sample contamination in the freezing of open carriers, there is no such report in the world at present, and some studies believe that whether open vitrification system or closed vitrification system have no significant impact on the freezing outcome of embryos (28). We have to admit that embryo freezing technology has helped countless infertile couples. In the future, whether for fertility preservation or for reducing OHSS risk, embryo freezing may be an increasing choice for clinicians. Therefore, perhaps we need more prospective research to explore which is safer, open, or closed vitrification system?

In conclusion, our study further confirmed patients undergoing immediate FET and delayed FET had comparable live birth rates and other pregnancy outcomes with a large amount of patients’ population. However, we failed to record reasons from patients why they chose immediate FET or delayed FET. And therefore, further high quality, randomized, controlled trials are needed to obtain more accurate results and conclusions.

## Data availability statement

The original contributions presented in this study are included in this article/supplementary material, further inquiries can be directed to the corresponding authors.

## Ethics statement

The studies involving human participants were reviewed and approved by the Ethics Committee of the Chengdu Jinjiang Hospital for Women’s and Children’s Health. The patients/participants provided their written informed consent to participate in this study.

## Author contributions

QW and M-XC designed the study and were responsible for the conception of the study and for manuscript drafting. X-JW, LT, H-JY, X-YL, Z-HZ, X-JT, and Y-BD contributed to the manuscript drafting and statistical analysis. YL and MX contributed to the revision and final approval of the manuscript. All authors contributed to the article and approved the submitted version.
